# RISK aversion in Italian forensic and non-forensic patients with schizophrenia spectrum disorders

**DOI:** 10.1371/journal.pone.0289152

**Published:** 2023-07-31

**Authors:** Nicola Canessa, Laura Iozzino, Sonia Andreose, Luca Castelletti, Giovanni Conte, Alexander Dvorak, Clarissa Ferrari, Janusz Heitzman, Ambra Macis, Inga Markiewicz, Giulia Mattavelli, Giuseppe Nicolò, Marco Picchioni, Giuseppe Restuccia, Gianfranco Rivellini, Fabio Teti, Giovanni de Girolamo

**Affiliations:** 1 IUSS Cognitive Neuroscience (ICON) Center, Scuola Universitaria Superiore IUSS, Pavia, Italy; 2 Istituti Clinici Scientifici Maugeri IRCCS, Cognitive Neuroscience Laboratory of Pavia Institute, Pavia, Italy; 3 IRCCS Istituto Centro San Giovanni di Dio Fatebenefratelli, Unit of Epidemiological Psychiatry and Evaluation, Brescia, Italy; 4 IRCCS Istituto Centro San Giovanni di Dio Fatebenefratelli, Psychiatric Unit, Brescia, Italy; 5 REMS del Veneto, ULSS9 Scaligera, Verona, Italy; 6 Department of Mental Health, ASST di Brescia, Brescia, Italy; 7 Justizanstalt Goellersdorf, Göllersdorf, Austria; 8 IRCCS Istituto Centro San Giovanni di Dio Fatebenefratelli, Unit of Statistics, Brescia, Italy; 9 Institute of Psychiatry and Neurology, Department of Forensic Psychiatry, Warsaw, Poland; 10 REMS Minerva, Department of Mental Health, ASL Roma 5, Rome, Italy; 11 Department of Forensic and Neurodevelopmental Science, Institute of Psychiatry, Psychology and Neuroscience, King’s College London, London, United Kingdom; 12 St Magnus Hospital, Surrey, United Kingdom; 13 REMS di Volterra, Azienda USL Toscana Nord Ovest, Volterra, Italy; 14 Sistema Polimodulare di REMS Provvisorie, ASST di Mantova, Castiglione delle Stiviere, Italy; Girne American University - Karmi Campus: Girne Amerikan Universitesi, CYPRUS

## Abstract

**Background:**

Goal-directed decision-making is a central component of the broader reward and motivation system, and requires the ability to dynamically integrate both positive and negative feedback from the environment in order to maximize rewards and minimize losses over time. Altered decision-making processes, in which individuals fail to consider the negative consequences of their decisions on both themselves and others, may play a role in driving antisocial behaviour.

**Aim:**

The main study aim was to investigate possible differences in loss and risk aversion across matched patients, all with a schizophrenia spectrum disorder (SSD), but who varied according to whether they had a history of serious interpersonal violence or not, and a sample of healthy controls with no history of violence.

**Results:**

The sample included 14 forensic and 21 non-forensic patients with SSD, and 41 healthy controls. Among the three decision-making variables under investigation, risk aversion was the only significant predictor of membership of the three groups, with greater risk aversion among non-forensic patients with SSD compared to healthy controls. No differences were observed across groups in loss aversion and choice consistency.

**Conclusions:**

This evidence suggests a new potential treatment target for rehabilitative measures aimed at achieving functional improvements in patients with SSD by selectively leveraging the neuro-cognitive processing of reward.

## 1. Introduction

Decision-Making is an inherent part of everyday-life, with effects at both individual and societal levels. Understanding the factors that influence decision-making is crucial for social and behavioural sciences, particularly when trying to identify the drivers of aberrant behaviour and violence. In Europe in 2019 there were 642,500 police-recorded assaults, up 11% from 577,400 in 2014 (https://ec.europa.eu/eurostat/web/crime/data/database). Such behaviours might reflect alterations in a key stage of decision-making such as mentally anticipating and evaluating the consequences of one’s own decisions and actions, particularly when they impact on another’s wellbeing.

From a biological perspective, this process involves the coordinated activity of a broad neural network. The fronto-limbic network and ventromedial prefrontal regions process appetitive and aversive stimuli [1–5], alongside the dorsal sector of the anterior cingulate cortex supporting outcome evaluation and performance monitoring [6], while the fronto-parietal network underpins executive control and behavioural adjustments [7–9]. Within this framework, individual variations in decision-making can be explained through differential sensitivity to ubiquitous economic trade-offs [10], such as those between probabilistic and certain outcomes, or between prospective gains and losses, and their association with psychological dimensions such as affect, arousal [11] and impulsivity [12]. While the former trade-off highlights *risk aversion* as the usual preference for certain, definite outcomes compared with probabilistic outcomes [10, 13], the concept of *loss aversion* reflects the larger psychological impact of anticipating negative, compared with positive, outcomes [4, 10, 14].

Different theoretical models have been proposed to explain the drivers of offenders’ behaviour [15], including the Routine Activity Theory [16], stressing the role of the situational context, or the Rational Choice Theory [17, 18], grounded in individual differences in processing the trade-off between risk versus certainty or immediate versus delayed outcomes. The effect of such variables on decision-making can be assessed using neuro-cognitive tasks capable of detecting differences across normal and pathological conditions that might explain real-life offending behaviour [7, 10]. Indeed, rates of mental disorders are higher among offenders than in the general population, with psychosis, substance use disorder and severe (psychotic) depression showing the highest rates [19, 20]. Despite preliminary evidence of impaired feedback-based learning in offenders [7], however, it is still unclear whether they differ from the general population in terms of risk taking [21–24].

Studies based on the Iowa Gambling Task (IGT) highlighted distinct patterns of impaired performance in criminal offenders compared with healthy controls [25], reflecting either hypersensitivity to rewards (i.e., overweighting of potential gains as compared to losses), or heightened sensitivity to immediate outcomes [18].

Importantly, most previous studies have investigated decision-making skills in offenders [24] and people with mental disorders (e.g., Hiser and Koenigs, 2018) [26] separately, thus, largely neglecting the possible combined effects of these factors on decision-making.

We therefore aimed to fill this gap by assessing risk taking in patients with schizophrenia spectrum disorders (SSD) with and without a history of serious forensic offending. Previous studies addressing risk taking in patients with SSD reported partially inconsistent evidence. Impaired IGT performance, in patients with SSD compared with healthy controls [27, 28], was found to reflect increased sensitivity to rewards [29] and the severity of depressive symptoms [30]. previous studies reported both increased [31, 32] and decreased [30, 33] risk aversion in patients with SSD compared to healthy controls, whereas an absence of patients’ loss aversion was reported in two studies [34, 35]. To date, however, well-established economic paradigms for testing loss and risk aversion within a single study [36] have been only employed in a non-clinical sample of untreated individuals displaying negative symptoms and/or hypomania [37], but not in SSD.

On this basis, we investigated possible differences in risk and loss aversion across sex- and age-matched patients with SSD with or without a history of serious interpersonal violence compared to healthy controls with no history of violence, while controlling for differences between the two patient groups in sex, lifetime substance/alcohol use, and education.

## 2. Materials and methods

### 2.1 Participants

This study was part of the EUropean VIOlence Risk and MEntal Disorders (EU-VIORMED), a European multicentre case-control study conducted in five countries (Austria, Germany, Italy, Poland and the UK) [38]. For organizational reasons (including issues related to the access to forensic facilities), this decision-making task was only administered to patients recruited in Italian forensic facilities.

The study included three groups: forensic patients with SSD, non-forensic patients with SSD and healthy controls. All patients had a confirmed diagnosis based on chart review of SSD according to DSM-5 (Diagnostic and Statistical Mental Disorders Manual) [39] criteria and were aged between 18 and 65 years. ‘Forensic’ patients were defined as patients with SSD that have committed a homicide, attempted homicide or other assault that caused serious physical injury to another person. They were recruited from forensic institutions.

‘Non-forensic’ patients had a SSD diagnosis, were sex- and age-matched to forensic patients, and had never committed an act of severe violence, as confirmed by medical records and by the treating clinicians. The task was also administered to a sample of healthy controls who had no history of mental disorder and no history of severe interpersonal violence.

Exclusion criteria for all groups included: (a) a diagnosis of intellectual disability; (b) a traumatic brain injury, an organic brain disorder or cancer; (c) inability to speak the national language fluently; and (d) planned discharge in the next month.

### 2.2 Recruitment

In each study centre, treating clinicians invited participants under their care to enter the study. Participants were provided with written information about the study and had an opportunity to ask questions. Informed consent was also sought to allow to collect information from caregivers, family members or case-managers/clinical staff for additional/missing information.

The recruitment of forensic cases was the priority, with care given to matching criteria (e.g., age categories, sex and diagnosis). This helped in finding matched non-forensic SSD participants, who were recruited from local adult psychiatric services.

The study was approved by the Ethical Committee of IRCCS Centro San Giovanni di Dio Fatebenefratelli (Brescia, Italy; permissions n. 74–2018 and n. 73–2019). All participants gave their written informed consent after a full description of the study aims and methods.

### 2.3 Socio-demographic, clinical, functional and violence assessment

All participants underwent a multidimensional standardized evaluation. A Patient Information Form (PIF) was used to collect socio-demographic and clinical information. The Index Violence Sheet (IVS) and a Risk Factors Questionnaire (RFQ) were *ad-hoc* instruments used to collect data about the index violence and violence risk factors for forensic patients (for more details see *Supporting Information*). The Positive and Negative Syndrome Scale-PANSS [40] was used to evaluate current psychotic symptoms [41, 42]. The Brief Assessment of Cognition in Schizophrenia (BACS) [43] evaluated cognitive functioning. The World Health Organization Disability Assessment Schedule (WHODAS-2.0) [44] was used to assess functioning and disability related to health conditions across six functional domains: cognition, mobility, self-care, getting along, life activities and participation.

### 2.4 Decision-making assessment

All participants performed a task designed to measure their degree of loss aversion (defined as the overweighting of negative, compared with positive, choice outcomes), and risk aversion (defined as the preference for certain compared to probabilistic outcomes). The former task included 49 trials in which the subjects had to choose between a certain “0” outcome and a lottery/gamble, which might result in a gain (G) or a loss (L) with equal (50–50%) probabilities. G and L changed at every trial, which allowed us to estimate the “loss aversion” (λ) parameter such that λ >1 or <1 indicates loss aversion or loss seeking, respectively. Importantly, the popular claim of an average λ≈2^15^ has been questioned by the evidence of no loss aversion for small to moderate amounts [45, 46]. Risk aversion was assessed with 30 further trials in which the participants were asked to choose between a certain gain (C) and a gamble which, with equal (50–50%) probabilities, might result in a gain G (with G > C) or nothing (0). C and G changed at every trial, which allowed to estimate a “risk aversion” (ρ) parameter reflecting the diminishing marginal sensitivity to probabilistic (i.e., risky) outcomes.

### 2.5 Statistical analyses: group comparisons

Frequencies and percentages for categorical variables, and means and standard deviations (SDs) for continuous variables, were evaluated. Chi-squared or Fisher’s exact tests were used to compare categorical variables among the groups of interest. The normality assumption for continuous variables distribution was tested through histogram plots and normality tests; coherently, group comparisons were performed by t-tests and ANOVA or the non-parametric Mann-Whitney and Kruskal-Wallis tests according to the nature of the data. Finally, multinomial logistic regression models were used to assess the associations between the three groups (dependent variable) and decision-making variables (independent variables), adjusting for potential confounders. All tests were two-tailed and the significance level was set at 0.05. The analyses were performed using IBM SPSS Statistics for Windows (Version 26.0. Armonk, NY: IBM Corp).

### 2.6 Extraction of decision-making parameters

Both gain-loss and gain-only trials were simultaneously fitted to the following Prospect-theory-inspired model [36]:

$$\mathrm{Pr}(accept\;gamble|G,L,B)=\frac{1}{1+e^{-\mu \times (p_{G}{\times (G)}^{\rho }-\lambda \times p_{L}\times {\left(-L\right)}^{\rho }-B^{\rho })}}$$

where G is the gain (G>0), L is the loss (L<0 for gain-loss gambles and L = 0 for gain-only gambles), B the guaranteed gain (B = 0 for gain-loss gambles and B>0 for gain-only gambles), pG = 0.5 is the probability of a gain and pL = 1-pG = 0.5 is the probability of a loss. The free parameters of the model are: a) the loss aversion lambda (λ), i.e., the multiplicative weight associated with anticipated losses compared with gains; b) the risk aversion rho (ρ), i.e., the curvature of the value function u(x) = x^ρ that embodies the diminishing sensitivity to increasing outcome; and c) the choice consistency or “softmax temperature” (μ), i.e., a measure of noisiness vs. systematicity in choices. They were estimated via maximum likelihood estimation with MATLAB (MathWorks, Natick, MA) for each subject separately.

## 3. Results

### 3.1 Sociodemographic and clinical characteristics

Of 116 subjects who expressed an initial interest in the study, 40 refused (25 patients, 62.5%, and 15 healthy controls, 37.5%); we were unable to collect further data on these subjects due to constraints posed by the Ethical Committee.

Following previous studies assessing loss aversion based on mixed-gambles [4, 14, 36], inspection of the individual responses led us to exclude participants if the model did not converge, suggesting either ‘random choice’ behaviour or that they misunderstood the instructions, and when choices were suggestive of the tendency to accept or reject all gambles [47, 48]. Moreover, since the lack of a real financial incentive did not allow to interpret a loss-seeking pattern, we retained loss-neutral participants (lambda = 1) and excluded those with lambda<1.

We thereby removed 15, 11 and 3 participants for lack of model convergence, lambda <1 and lambda >8, respectively. Thus, the overall sample included 14 forensic and 21 non-forensic patients with SSD, and 41 healthy controls.

Table 1 shows the socio-demographic characteristics of both forensic and non-forensic patients and healthy controls. The majority of participants were males (N = 51; 67.1%), with a further male excess in the clinical group (forensic and non-forensic patients) compared to healthy controls (p<0.001).

**Table 1:** Socio–demographic characteristics of forensic patients with SSD, non–forensic patients with SSD and healthy controls.

	Forensic group (FG, N = 14) N (%)	Non-Forensic group (NFG, N = 21) N (%)	Healthy controls Group (HC, N = 41) N (%)	p-value	Post-hoc
**Sex**					
*Male*	13 (92.9)	19 (90.5)	19 (46.3)	<0.001	
*Female*	1 (7.1)	2 (9.5)	22 (53.7)		
**Age**, Mean (SD)	39.9 (12.8)	38.3 (12.7)	40.3 (15.8)	0.864	
**Age**					
*18–29*	3 (21.4)	7 (33.3)	15 (36.6)	0.538	
*30–41*	6 (42.9)	5 (23.8)	9 (22.0)		
*42–53*	1 (7.1)	6 (28.6)	8 (19.5)		
*54–69*	4 (28.6)	3 (14.3)	9 (22.0)		
**Marital status** *					
*Married or cohabiting*	1 (7.1)	3 (14.3)	19 (50.0)	0.001	
*Single*	10 (71.4)	18 (85.7)	16 (42.1)		
*Divorced or widowed*	3 (21.4)	0 (0.0)	3 (7.9)		
**Education years**, Mean (SD)	9.9 (3.9)	10.8 (2.5)	15.7 (4.3)	<0.001	FG vs HC <0.001 NFG vs HC <0.001
**Highest occupational status**					
*Never worked/ Student/housewife*	2 (14.3)	2 (9.5)	5 (12.2)	<0.001	
*Unskilled worker*	11 (78.6)	14 (66.7)	5 (12.2)		
*Skilled worker*	1 (7.1)	5 (23.8)	22 (53.7)		
*Professional*	0 (0.0)	0 (0.0)	9 (22.0)		
**Lifetime substance/alcohol use**					
*Yes*	10 (71.4)	10 (47.6)	1 (2.4)	<0.001	
*Never*	4 (28.6)	11 (52.4)	40 (97.6)		

*Frequencies and percentages, as well as means and standard deviation, have been evaluated considering only the valid cases (i.e., all the cases with no missing data). Chi squared or Fisher’s exact tests (when expected counts <5 in at least one cell) have been performed for categorical variables; ANOVA and Bonferroni post–hoc corrections have been performed for Education years.

Compared to forensic and non-forensic patients with SSD, healthy controls were more frequently married or cohabiting (p = 0.001), had achieved a higher educational level (p<0.001) and were more often skilled or professional workers (<0.001). Moreover, forensic and non-forensic patients with SSD more often had a lifetime history of substance and alcohol misuse compared to healthy controls (p<0.001).

Descriptive statistics and between-groups comparisons regarding clinical characteristics in forensic and non-forensic patients are shown in Table 2. The most common diagnosis in both groups was schizophrenia (85.7%). Mean age at first contact with psychiatric services differed significantly between the two groups (p = 0.029), while the mean duration of illness was over 7 years in both groups. Forensic patients were more likely to meet criteria for a comorbid personality disorder than non-forensic patients (p = 0.006). There were no significant differences in current positive (p = 0.210) and negative (p = 0.881) symptoms, general psychopathology (p = 0.934) and PANSS total scores between the two patient groups (p = 0.778).

**Table 2:** Clinical characteristics of forensic patients with SSD and non–forensic patients with SSD.

	Forensic group (N = 14) N (%)	Non-Forensic group (N = 21) N (%)	p-value
**Illness duration (Years), Mean (SD)** *	7.8 (5.0)	13.4 (12.2)	0.076
**Age of first contact with DMHs (Years), Mean (SD)**	32.5 (10.3)	25.3 (8.2)	**0.029**
**Type of SSD diagnosis**			
*Schizophrenia*	9 (64.3)	14 (66.7)	**0.034**
*Schizoaffective disorders*	1 (7.1)	7 (33.3)	
*Delusional disorder*	1 (7.1)	0 (0.0)	
*Brief psychotic disorder*	1 (7.1)	0 (0.0)	
*Schizophreniform disorder*	1 (7.1)	0 (0.0)	
*Drug-induced psychosis*	1 (7.1)	0 (0.0)	
**Comorbidity with Personality disorder**s			
*No*	9 (64.3)	21 (100.0)	**0.006**
*Yes*	5 (35.7)	0 (0.0)	
**Antipsychotics**			
*No*	0 (0.0)	3 (14.3)	0.259
*Yes*	14 (100.0)	18 (85.7)	
**PANSS**			
*Positive symptoms*	12.1 (4.3)	14.0 (4.9)	0.210
*Negative Symptoms*	16.6 (5.0)	17.7 (6.8)	0.881
*General psychopathology*	30.6 (5.3)	31.5 (8.4)	0.934
*Total score*	59.3 (9.2)	63.1 (17.4)	0.778
**WHODAS 2.0**			
*Total score* *	1.6 (2.4)	8.1 (8.4)	**0.006**
**BACS**			
*List learning* *#*	35.6 (8.5)	37.4 (10.7)	0.727
*Digits sequencing task* *#*	15.1 (4.7)	16.4 (5.7)	0.454
*Token motor task* *#*	52.4 (13.2)	51.5 (16.3)	0.654
*Verbal fluency* *#*	34.5 (12.8)	39.1 (14.6)	0.396
*Symbol coding task* *#*	33.0 (18.6)	41.4 (15.6)	0.164
*Tower of London* * *#*	14.6 (2.8)	15.9 (4.1)	0.071

PANSS: Positive and Negative Syndrome Scale; WHODAS 2.0: World Health Organization Disability Assessment Schedule 2.0; BACS: Brief Assessment of Cognition in Schizophrenia.

*Means and standard deviations have been evaluated considering only valid cases (i.e., all cases with no missing data). Available data: Illness duration = 34/35; WHODAS = 32/35; BACS Tower Of London = 34/35.

#Raw scores

Chi squared or Fisher’s exact test (when expected counts <5 in at least one cell) has been performed for categorical variables; non parametric Kruskal–Wallis test has been performed for all PANSS scores, for WHODAS 2.0 overall score and for all BACS scores; t–test has been performed for Illness duration and Age of first contact with DMHs.

Forensic patients displayed better social functioning and lower level of disability, as assessed with the WHODAS (mean score: 1.6, SD = 2.4 for the forensic group vs mean score: 8.1, SD = 8.4 for the non-forensic one; p = 0.006).

Finally, forensic and non-forensic patients did not differ in terms of their neuro-cognitive parameters as assessed using the BACS.

### 3.2 Decision-making

There was a significant difference between the three groups in terms of risk aversion (p = 0.019). Post-hoc comparisons showed that this difference was driven by greater risk aversion among non-forensic patients with SSD compared to healthy controls (p = 0.020), with Cohen’s d = 0.69 suggesting a medium effect size. There were no significant differences among the three groups in loss aversion (p = 0.558) and choice consistency (p = 0.389) (Table 3).

**Table 3:** Decision–making experiment of forensic patients with SSD, non–forensic patients with SSD and healthy controls.

	Forensic group (FG, N = 14) Mean/Median (SD)	Non-Forensic group (NFG, N = 21) Mean/Median (SD)	Healthy controls (HC, N = 41) Mean/Median (SD)	p-value	post-hoc	Cohen’s d
**Loss aversion**	1.9/1.88 (0.8)	2.3/1.88 (1.1)	2.3/2.01 (1.4)	0.558		
**Risk aversion**	1.0/0.8 (1.1)	0.5/0.4 (0.5)	0.8/0.8 (0.4)	**0.019**	(NFG) vs (HC) **0.020**	0.69
**Consistency**	2.7/1.1 (3.3)	2.0/1.1 (2.8)	1.2/0.6 (1.5)	0.389		

Non parametric Kruskal–Wallis test has been performed.

These findings were also confirmed by multinomial logistic models adjusted for sex, lifetime substance/alcohol use and education (Table 4): although the corresponding odds ratios were not significant, risk aversion was the only significant predictor of membership of the three groups (p = 0.028) among the three decision-making variables investigated. This significant effect is due to the difference between the non-forensic group and healthy controls.

**Table 4:** Association between group and decision–making variables.

Model	Independent variable	p-value overall effect	OR (p-value)
**A**	**Loss aversion**	0.181	
	FG vs HC		0.46 (0.105)
	NFG vs HC		0.77 (0.356)
	**Education**	**0.003**	
	FG vs HC		0.72 (0.016)
	NFG vs HC		0.73 (0.005)
	**Lifetime Substance/Alcohol use (ref = Yes)**	**0.001**	
	FG vs HC		0.01 (0.017)
	NFG vs HC		0.02 (0.051)
	**Sex (ref = Female)**	**0.042**	
	FG vs HC		5.22 (0.195)
	NFG vs HC		7.22 (0.032)
**B**	**Risk aversion**	**0.028**	
	FG vs HC		1.78 (0.557)
	NFG vs HC		0.27 (0.153)
	**Education**	**0.002**	
	FG vs HC		0.68 (0.005)
	NFG vs HC		0.76 (0.013)
	**Lifetime Substance/Alcohol use (ref = Yes)**	**0.002**	
	FG vs HC		0.01 (0.009)
	NFG vs HC		0.03 (0.037)
	**Sex (ref = Female)**	**0.050**	
	FG vs HC		4.91 (0.209)
	NFG vs HC		6.83 (0.036)
**C**	**Uncertainty**	0.512	
	FG vs HC		1.25 (0.269)
	NFG vs HC		1.16 (0.440)
	**Education**	**0.001**	
	FG vs HC		0.69 (0.005)
	NFG vs HC		0.73 (0.004)
	**Lifetime Substance/Alcohol use (ref = Yes)**	**0.003**	
	FG vs HC		0.02 (0.009)
	NFG vs HC		0.04 (0.037)
	**Sex (ref = Female)**	**0.051**	
	FG vs HC		5.01 (0.194)
	NFG vs HC		6.72 (0.037)

FG = Forensic Group; NFG = Non-Forensic Group; HC = Healthy Controls.

Multinomial logistic models were adjusted for sex, lifetime substance/alcohol use and education. The reference category of the dependent variable (Group) is HC.

## 4. Discussion

To the best of our knowledge, this is the first study which used well-established behavioural economics paradigms to compare loss and risk aversion across patients with SSD with and without a history of serious violence, and healthy participants. A multi-domain assessment of patients’ cognitive status was performed using the BACS, that was able to exclude group differences in cognition as contributing to differential decision-making performance.

There were no significant differences in any decision-making variables between forensic and non-forensic patients with SSD, while higher risk aversion was observed in non-forensic patients with SSD compared with healthy controls.

### 4.1 Risk aversion in people with SSD

This new evidence provides novel insights into a controversial literature showing both increased [31, 32] and decreased [33, 34] risk aversion in patients with SSD. Unlike some of these earlier studies, administering both gain-loss and gain-only trials allowed us to disentangle the effects of anticipating losses from that associated with risk in itself, resulting in a more specific assessment of risk aversion. This approach confirmed a more conservative decision-making in patients with SSD compared to healthy controls, which, alongside the lack of differences in loss aversion, suggests an impaired reward-based learning, with preserved punishment-based learning, in patients with SSD [31].

While our conclusions are limited by the small sample size, the present evidence of a significant difference in risk aversion suggests that our negative finding for differential loss aversion in SSD patients cannot be merely ascribed to power issues. Moreover, altered loss aversion in people with SSD is an issue for debate: previous related reports either found loss aversion to be absent in patients with SSD [34, 35] or, conversely, found no significant difference across patients and controls in this economic dimension [33]. Importantly, however, the studies reporting no differences in SSD inferred loss aversion from differential gain/loss scenarios within tasks assessing interpersonal coordination in a Prisoner’s dilemma [34] or price estimation in selling vs. buying conditions [35]. Both these experimental paradigms largely depart from those used in the psycho-economic literature, where individual differences in loss/punishment sensitivity are assessed through tasks that require a choice between accepting or rejecting mixed-gambles, thereby allowing direct tracking of the economic and affective weight placed on prospective losses [49, 50]. It is thus noteworthy that the present evidence of no significant differences between patients with SSD and healthy controls fits with that reported in the only previous study using mixed gambles to assess loss aversion in patients with SSD [33]. Overall, these findings suggest greater difficulties for SSD patients in implementing positive rather than negative feedback, when learning from past experiences.

In turn, this evidence suggests a new potential treatment target for rehabilitative measures aimed at achieving functional improvements in patients with SSD by selectively leveraging the neuro-cognitive processing of reward. Our results show that the greater risk aversion observed in patients with SSD is at least partially explained by education levels and past substance/alcohol use. While contributing to a controversial literature on the effects of alcohol intoxication [51, 52] and lifetime alcohol use [53, 54] on risk taking, these findings additionally suggest that socio-demographic considerations (such as the educational level or the marital status) should be evaluated when comparing the processing of risky outcomes across different populations.

### 4.2 Risk aversion in forensic and non-forensic patients with SSD

The lack of differences between forensic and non-forensic patients in our sample contributes to a controversial literature that has reported both greater risk-seeking levels [55], or normal adjustments of risk-taking to outcome probability [22, 23], in offenders. Previous studies reported alterations of typical “cool” executive functions (e.g., response-inhibition or planning) in this population, with no involvement of their “hot” (i.e., affect- or reward-related) counterpart, such as reversal-learning [21, 56]. The latter finding fits with previous evidence of comparable impulsivity and risk-taking in the Cambridge Gambling task [21]. By complementing these negative findings through a well-validated experimental paradigm, our results suggest that the cognitive alterations associated with forensic behaviour either involve cool executive functions (e.g., response inhibition) more than reward or risk valuations, or they are not properly captured by the decision-making tasks used in these studies. Indeed, the few studies reporting greater risk-taking in offenders have generally used the Iowa gambling task [57], which requires the coordinated activity of both hot and cool executive functions [57]. Moreover, the selective observation of altered risk aversion in non-forensic patients with SSD fits with recent evidence of greater risk aversion in this disorder, but not in SSD patients with anti-social personality disorders [32]. Altogether, these results might suggest that—if offenders’ forensic behaviour reflects alterations in specific valuation and decision-making processes–these might involve aspects other than loss or risk aversion, or that these economic dimensions should be assessed through choices involving other individuals’ welfare [58].

### 4.3 Limitations

Due to constraints posed both by the Ethical Committee and the specific treatment setting where the study took place (e.g., forensic facilities), we were not allowed to incentivize participants. This is clearly a limitation in a study on decision-making, particularly because it hampers interpreting values of lambda close to 1, i.e., those distinguishing among loss-averse, -neutral- and -seeker participants. This is a critical issue in the current literature on loss aversion, since the popular claim of an average λ≈2^15^ has been questioned by the evidence of no loss aversion for small to moderate amounts [45, 46]. Based on the above considerations, we decided to exclude participants with lambda<1 (since their loss-seeking behaviour would not be interpretable in the lack or real losses), while retaining those will lambda = 1 (and thus accommodating a loss-neutral pattern). The lack of real financial outcomes might have thus biased the present findings, which are therefore in need of replications by future studies with larger samples and a more ecological incentivization procedure.

## 5. Conclusions

This is the first study to compare patients with SSD with and without a history of serious violence, and healthy controls, in a well-validated risk-taking behavioural paradigm. Recruiting non-forensic patients with SSD as a primary control group allowed us to better unpick the effects of mental disorders and other clinically-relevant factors which might otherwise contribute to seemingly antisocial behaviour. Despite the lack of significant evidence for selective neuro-cognitive markers of offending, we identified aspects of decision-making under risk which might prove useful for assessing altered high-order executive functioning in people with SSD. While further studies are required to investigate whether, and to what extent, such alterations might also help explain antisocial behaviours, such as those displayed by forensic sample, these findings highlight specific target cognitive domains for treatment and rehabilitation in patients with SSD.

## Supporting information

S1 File Index Violence Sheet–IVS. (DOCX)

S2 File Questionnaire on risk factors for the index violence–Q. (DOCX)

S3 File Full instructions of the risk aversion task. (DOCX)

S4 File Full instructions of the loss aversion task. (DOCX)
