# Supplementary material for: RISK aversion in Italian forensic and non-forensic patients with schizophrenia spectrum disorders
Source: PLoS One. 2023 Jul 31;18(7):e0289152. doi: 10.1371/journal.pone.0289152 (PMC10389697; doi:10.1371/journal.pone.0289152)
Supplement: S1 File — (DOCX) [file pone.0289152.s001.docx]

**
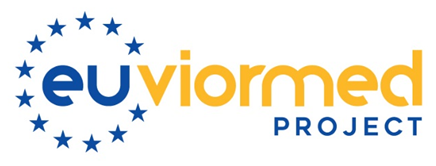

Supporting information - 1**

**Index Violence* Sheet – IVS**

**For cases only**

**** Index Violence refers to the violent offence used for recruitment purposes***

**Examiner:** ________________________________________

**IVS_a_1 Date form completed (dd/mm/yyyy):** __________________

| *code:* | **0** | **1** | **2** | **3** | **4** | **5** |
| --- | --- | --- | --- | --- | --- | --- |
| **IVS_a_2 Data source:** | Clinical charts/ clinicians | Family members | Patient | Both the patient and clinicians | Both the patient and family members | All – patient, clinicians and family members |

|  |
| --- |

**IVS_01** *Type of crime (index violence):*

Homicide code=0

Attempted homicide code=1

Assault with injury code=2

Used a weapon, force, or intimidation to get money or things from a person code=3

Other code=4

|  |  |  |  |
| --- | --- | --- | --- |

**IVS**_**02** *Date – when the index violence occurred (yyyy)*

Not known code=MISS

|  |
| --- |

**IVS_03** *Type of index violence*

Reactive/impulsive code=0

Premeditated code=1

Not known code=MISS

|  |
| --- |

**IVS_04** *History of conviction or arrest for the index violence*

No code=0

Yes (current forensic unit) code=1

Yes (another forensic unit ) code=2

Yes (jail) code=3

Not known code=MISS

|  |
| --- |

**IVS_05** *Victim(s) of the index violence*

Family – parents code=0

Family other including partner code=1

Friends code=2

Clinical staff or other patients code=3

Acquaintances code=4

Strangers code=5

Not known code=MISS

|  |
| --- |

**IVS_06** *Main* *consequences for the victim*

Death code=0

Severe injuries code=1

Mild injuries code=2

|  |
| --- |

**IVS_07** *Patient’s intent when committing index offence*

Clear instrumental intent to harm code=0

Self-defence code=1

Obeying command code=2

Involvement in a criminal gang code=3

Unclear/unknown code=MISS

|  |
| --- |

**IVS_08** *Was the patient very angry at time of index offence?*

No code=0

Yes, reported in clinical reports code=1

Yes, reported by the patient code=2

Not known code=MISS
