# Supplementary material for: RISK aversion in Italian forensic and non-forensic patients with schizophrenia spectrum disorders
Source: PLoS One. 2023 Jul 31;18(7):e0289152. doi: 10.1371/journal.pone.0289152 (PMC10389697; doi:10.1371/journal.pone.0289152)
Supplement: S2 File — (DOCX) [file pone.0289152.s002.docx]

**
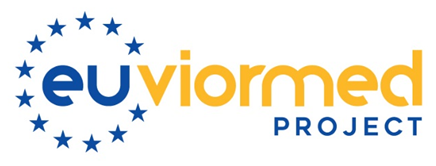
**

# Supporting information – 2

# Questionnaire on risk factors for the index violence*** – Q

# For cases only

**** Index violence refers to the violent offence used for recruitment purposes***

**Q_a_1 Date form completed (dd/mm/yyyy):** __________________

**Examiner:** ________________________________________

| *code:* | **0** | **1** | **2** | **3** | **4** | **5** |
| --- | --- | --- | --- | --- | --- | --- |
| **Q_a_2 Data source:** | Clinical charts/ clinicians | Family members | Patient | Both the patient and clinicians | Both the patient and family members | All – patient, clinicians and family members |

**PATIENT’S CHARACTERISTICS AT TIME OF THE INDEX VIOLENCE**

**Q_01** *Unemployed*

No code=0

Yes code=1

Not known code=MISS

**Q_02** Site of **FIRST** *admission under legal order (i.e., sectioned or mandated) as a consequence of the index violence*

Forensic Unit code=0

Prison code=1

Not known code= MISS

**Q_02a** *Specific section/criminal code act which the patient is CURRENTLY detained under*

(txt):_________________________________________________________________________________________

Not know code=MISS

**Q_03** *Evidence of poor self-care (at time of the index violence)*

No code=0

Yes code=1

Not known code= MISS

**Q_04** *Abnormal mental state at time of index violence according to the clinical records?*

No code=0

Yes code=1

Not known code= MISS

**Q_04a** *If YES at Q_04, please specify*

Psychotic disorder code=0

Mood disorder code=1

Other code= 2

Not known code= MISS

**Q_05** *Substance or alcohol misuse at time of index violence (for example, alcohol use exceeding recommended limits = intoxication or features of alcohol dependence)*

No code=0

Yes code=1

Not known code= MISS

**Q_06** *Previous hospitalization(s) for mental illness (before the index violence)*

No code=0

Yes code=1

Not known code= MISS

**Q_07** *Already in contact with mental health services before index violence*

No code=0

Yes code=1

Not known code= MISS

**Q_07a** *If YES at Q_07, diagnosis established before index violence*

No code=0

Yes, psychotic disorder code=1

Yes, personality disorder code=2

Yes, mood disorder code=3

Yes, anxiety disorder code=4

Yes, substance/alcohol disorder code=5

Not known code= MISS

**Q_07b** *If Yes at Q_07, Psychotropic medications at time of index violence*

Antipsychotic code=0

Antidepressant code=1

Mood stabilizer code= 2

Anxiolytic code= 3

Not known code= MISS

**Q_07c** If Yes at Q_07, Patient’s compliance to medication at time of index violence

No code=0

Yes code=1

Not known code= MISS
