# Supplementary material for: RISK aversion in Italian forensic and non-forensic patients with schizophrenia spectrum disorders
Source: PLoS One. 2023 Jul 31;18(7):e0289152. doi: 10.1371/journal.pone.0289152 (PMC10389697; doi:10.1371/journal.pone.0289152)
Supplement: S3 File — (DOCX) [file pone.0289152.s003.docx]

**Supporting information – 3**

**Full instructions of the risk aversion task**

*This task includes several trials involving financial outcomes. In every trial you will be asked to choose between:*

*a) a gamble presented in the left part of the sheet, or*

*b) a sure outcome presented in the right part of the sheet.*

*If you choose the gamble, you will have 50% probability of winning the amount shown in the superior part of the sheet, but also 50% probability of winning nothing. If you choose the option on the right, instead, you will have the certainty of winning the reported outcome. Your task is to choose which option you prefer, by selecting as fast as possible either the "left" or "right" option.*

*Please evaluate each trial and make your decision as if these outcomes were real.*
